# Quantitative mass spectrometry of TATA binding protein-containing complexes and subunit phosphorylations during the cell cycle

**DOI:** 10.1186/1477-5956-7-46

**Published:** 2009-12-24

**Authors:** WWM Pim Pijnappel, Annemieke Kolkman, Marijke PA Baltissen, Albert JR Heck, HT Marc Timmers

**Affiliations:** 1 Netherlands Proteomics Centre, Department of Physiological Chemistry, University Medical Center Utrecht, Universiteitsweg 100, 3584 CG Utrecht, the Netherlands; 2 Netherlands Proteomics Centre, Biomolecular Mass Spectrometry and Proteomics Group, Bijvoet Center for Biomolecular Research and Utrecht Institute for Pharmaceutical Sciences, Utrecht University, Faculty of Science, Padualaan 8, 3584 CH Utrecht, the Netherlands

## Abstract

**Background:**

Progression through the cell cycle is accompanied by tightly controlled regulation of transcription. On one hand, a subset of genes is expressed in a cell cycle-dependent manner. On the other hand, a general inhibition of transcription occurs during mitosis.

Genetic and genome-wide studies suggest cell cycle regulation at the level of transcription initiation by protein complexes containing the common DNA-binding subunit TATA binding protein (TBP). TBP is a key player in regulating transcription by all three nuclear RNA polymerases. It forms at least four distinct protein complexes with TBP-associated factors (TAFs): SL1, B-TFIID, TFIID, and TFIIIB. Some TAFs are known to remain associated with TBP during the cell cycle. Here we analyze all TAFs and their phosphorylation status during the cell cycle using a quantitative mass spectrometry approach.

**Results:**

TBP protein complexes present in human cells at the G2/M and G1/S transitions were analyzed by combining affinity purification with quantitative mass spectrometry using stable isotope labeling with amino acids in cell culture (SILAC). Phosphorylations were mapped and quantified after enrichment of tryptic peptides by titanium dioxide. This revealed that subunit stoichiometries of TBP complexes remained intact, but their relative abundances in nuclear extracts changed during the cell cycle. Several novel phosphorylations were detected on subunits of the TBP complexes TFIID and SL1. G2/M-specific phosphorylations were detected on TAF1, TAF4, TAF7, and TAFI41/TAF1D, and G1/S-specific dephosphorylations were detected on TAF3. Many phosphorylated residues were evolutionary conserved from human to zebrafish and/or drosophila, and were present in conserved regions suggesting important regulatory functions.

**Conclusions:**

This study provides the first quantitative proteomic analysis of human TBP containing protein complexes at the G2/M and G1/S transitions, and identifies new cell cycle-dependent phosphorylations on TAFs present in their protein complex. We speculate that phosphorylation of complex-specific subunits may be involved in regulating the activities of TBP protein complexes during the cell cycle.

## Background

Gene transcription is regulated during the cell cycle. During mitosis, transcription by all three nuclear RNA polymerases (pols) is inhibited [1,2]. In addition, 500-1000 genes are preferentially expressed at a particular stage of the cell cycle [3,4]. The regulation of cell cycle-dependent gene expression can occur at one of several levels. As a result of complex networks of kinases and phosphatases, the activities of sequence specific transcription factors like E2F, B-Myb, and FOXM1 can be modulated [5-7]. Another level of cell cycle regulation is chromatin, which can occur both at the level of chromatin remodeling, histone modification, and modification-specific chromatin association (reviewed in [8]). A third level of regulation of cell cycle-dependent gene expression is the basal transcription machinery. Transcription initiation by the three RNA polymerases is regulated by distinct protein complexes including those containing the common subunit TBP (TATA binding protein) and complex-specific TAFs (TBP associated factors) (reviewed in [9-12]). These are in human cells: the SL1 complex (with TAF1A-C and JOSD3/TAFI41/MGC5306/TAF1D, hereafter referred to as TAFI41/TAF1D) for pol I transcription; TFIID (with TAF1-13) and B-TFIID (with BTAF1) for pol II transcription; and TFIIIB (with Brf1 and the loosely associated Bdp1 protein) for pol III transcription. Genetic and genome-wide functional analyses point to specific cell cycle functions of the TFIID TAFs. Studies in yeast have identified temperature sensitive mutations in several TAF genes which result in cell cycle arrest at either G1 or G2/M, and include the TAF1, TAF2, TAF5, and TAF10 genes [13-15]. Mammalian TFIID TAFs also have cell cycle roles. Murine F9 embryonal carcinoma cells lacking TAF10 arrest at G1 and undergo apopotosis [16]. A genetic screen for genes required for cell cycle progression in hamster cells identified TAF1 as cell cycle regulated gene 1 (CCG1) involved in G1 progression [17]. Genome-wide RNAi screens have identified TAF4 and TAF13 to be important for G1 progression [18]. This study also identified the preinitiation complex factors TFIIB and TFIIEβ, which are recruited in response to TFIID promoter binding, to be important for G1 progression. In addition, the TFIIIB subunit Brf1 has been functionally linked to the cell cycle as its levels were found to be important for cell proliferation and oncogenic transformation, which seems mediated by tRNA^met ^levels [19].

Functional studies have mainly focused on the mechanisms underlying mitotic inhibition of transcription. These led to several models in which phosphorylation on TAFs regulate their activities. For SL1 mediated transcription, phosphorylation on TAF1C at T852 by cdk1/cyclin B has been implicated in mitotic inhibition [1]. Reactivation of transcription in G1 involved phosphorylation of the SL1 interactor UBF at S388 and S484 [20]. TFIID mediated transcription was marked by a mitotic hyperphosphorylation of TAF12, and phosphorylated TFIID showed decreased *in vitro *transcription activity [21]. This study also showed that several interactions within mitotic TFIID remained present. The TFIIIB subunit Brf1 is also subject to mitotic phosphorylation, which has been reported to lead to transcriptional repression and release of its interactor Bdp1 from chromatin [22,23].

Thus, phosphorylation on TAFs seems to be an important mechanism for mitotic inhibition of basal transcription factors. Far less information is available on the role of general transcription factors in regulating cell cycle specific patterns of gene expression. It is possible that changes in the phosphorylation status of individual TAFs regulate TBP complex function at cell cycle dependent genes. Another mechanism may be formation of TFIID subcomplexes with distinct functions, such as the Small TAF Complex consisting of TAF8, TAF9, and SPT7L [24,25]. However, it is unknown whether TBP subcomplexes have specific roles during the cell cycle.

The importance of TBP and TAFs for cell cycle progression prompted us to address the question whether protein complex formation around TBP is cell cycle regulated. We applied a quantitative mass spectrometry approach combined with affinity purification. This allowed us to determine changes in complex composition and relative abundances. In addition, novel phosphorylations present on TAFs in the TBP complexes SL1 and TFIID were identified and quantified by SILAC. Our results indicate that both the abundances in cell extracts and subunit phosphorylations are cell cycle regulated, while the compostions of TBP complexes remain unaffected. These findings present the first quantitative proteomic analysis of TBP complexes during the cell cycle, and offer new prospects on functional studies of transcriptional regulation during the cell cycle.

## Results and discussion

### Quantification of cell cycle-dependent TBP-containing protein complexes

**Figure 1:**
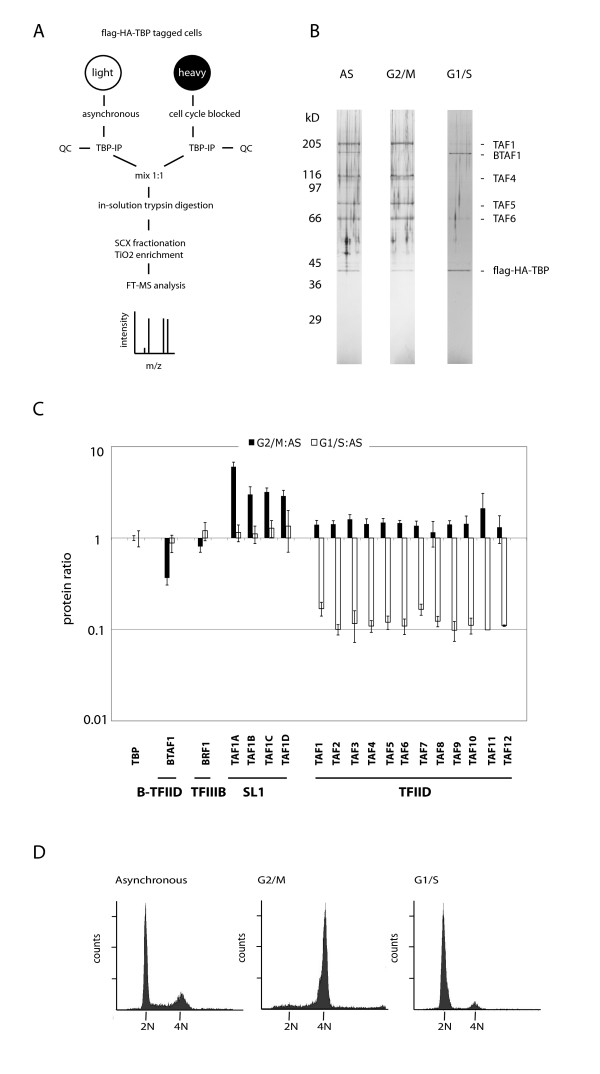
**Quantitation of TBP interactors in mitotic and S phase cells**. (A) Experimental setup. TBP complexes were isolated from SILAC-labelled cells grown asynchronously or blocked at G2/M or G1/S. QC: ~2% of the immunoprecipitates were analyzed on NuPage 4-12% Bis-Tris gradient gel followed by silver staining for quality control, as shown in (B). The rest of the immunoprecipitates were digested in solution with trypsin. Tryptic fragments were fractionated by SCX and phosphopeptides were enriched using titanium dioxide. Peptides were identified and quantified by nano-LC-MS. (C) Quantitation of TBP interactors in G2/M blocked or G1/S blocked cells relative to asynchronous cells. Protein levels are normalized for TBP. Data represent means of at least three quantified peptides +/- SD. (D) FACS profiles of asynchronous (left panel), nocadozole-treated (middle panel), and double thymidine-treated (right panel) cells.

To investigate whether TBP-containing protein complexes are subjected to cell cycle-dependent alterations, we used a combination of double affinity purification and quantitative mass spectrometry. This procedure is outlined in Fig 1a. Stable HeLaS3 cells expressing flag-HA tagged TBP at near endogenous levels were used. These cells were generated by retroviral transduction using pBabe-puro-N-flag-HA-TBP. After selection with puromycin, individual clones were picked, expanded and analyzed by immunoblot analysis using antibodies against flag, HA, and TBP (data not shown). This identified several clones with subendogenous expression of tagged TBP, one of which was used in this and our previous work [26]. The flag-HA epitope tags allowed double affinity purification of protein complexes using antibody-based resins and epitope peptide elutions. To obtain sufficient quantities, cells were grown in 15 liter-size bioreactors resulting in yields of 5-10 × 10^9 ^cells. We compared asynchronous (AS) cultures with G2/M-enriched (blocked using nocodazole) or G1/S-enriched (blocked using double thymidine) cultures and used SILAC to allow quantitation by mass spectrometry [27,28]. SILAC was performed by metabolic labeling of cells either with 'light' versions of arginine and lysine (^12^C_6_,^14^N_4_-arginine and ^12^C_6_,^14^N_2_-lysine) or with 'heavy' versions of these amino acids (^13^C_6_,^15^N_4_-arginine and ^13^C_6_,^15^N_2_-lysine). FACS analysis of propidium iodide-stained cells indicated that thymidine-treated cells were blocked at G1 just before S-phase, and showed an absence of cells with a DNA content indicative of cells in S-phase (Fig 1d). Nocadozole-treated cells were blocked at G2/M. Release experiments followed by FACS analysis confirmed cell cycle blocks as G2/M-blocked cells proceeded to G1, while G1/S-blocked cells proceeded to S-phase (data not shown). Whole cell extracts from the two conditions were separately purified, and subsequently mixed, concentrated, and analyzed by mass spectrometry. Because the samples were mixed after purification, and not before, potential exchange between the two samples during the affinity purification procedure was prevented. This was particularly important because we recently showed that BTAF1 is a highly dynamic complex subunit that dissociates and reassociates with TBP during immunopurification [26]. 2% of the immunoprecipitates were analyzed on silver stained gels for quality control (QC). This analysis revealed the TBP interactors: TAF1, BTAF1, TAF4, TAF5, TAF6, and tagged TBP itself (Fig 1b). These proteins seemed to change little at G2/M, while the TFIID-specific TAFs TAF1, TAF4, TAF5, and TAF6 seemed to be present at lower amounts relative to the B-TFIID-specific BTAF1 and tagged TBP at G1/S (Fig 1c). The samples were analyzed by in-solution tryptic digestion followed by SCX fractionation, titanium oxide enrichment of phosphopeptides [29], and nano-LC-MS. This confirmed and extended the results obtained by analysis of silver stained gels.

On beforehand, the SILAC/titanium dioxide enrichment approach allows alterations in the detection of missing stable subunits, changes in relative abundances of TBP complexes, and cell cycle-dependent phosphorylations. Each of these events would be reflected by specific changes in the SILAC ratios after normalization relative to the abundance of TBP peptides. Missing stable subunits would be detected by strongly reduced SILAC ratios (relative to asynchronous). Our results indicate that all subunits of SL1 and TFIID remained complexed with TBP, both in G2/M- and in G1/S-arrested cells, as all subunits of these complexes clustered together (Fig 1c; see Additional File 1 for sequence coverage). No evidence was obtained for changes of specific TBP-subcomplexes. Changes in relative complex abundance were detected by changed SILAC ratios for all subunits of the TFIID complex. Fig 1c showed increased SILAC ratios for all TFIID subunits in G2/M (relative to non-synchronized), while SILAC ratios for these TAFs were reduced in G1/S cells. This suggested that the abundance of TFIID in these extracts was subject to cell cycle regulation. The abundance of B-TFIID was decreased in extracts derived from G2/M cells, but not in G1/S derived extracts. The abundance of SL1 was increased at G2/M, but not at G1/S, while the TFIIIB subunit BRF1 showed unchanged associations at G2/M and G1/S. We would like to point out that we have used a mild salt extraction protocol [30]) in order to maintain TBP complex integrity. Importantly, re-extraction of remaining chromatin pellets with higher salt solubilised a significant proportion of TBP complexes (data not shown). The total amount of cellular TAFs and TBP is not modulated during the cell cycle. This suggests that chromatin association of TBP complexes may be subject to cell cycle regulation. Therefore, we conclude that data on the abundances of TBP complexes in cellular extracts during the cell cycle should be interpreted with caution. Our SILAC approach indicates that TBP complexes remain intact at the G2/M and G1/S transitions, and that B-TFIID, SL1, TFIID but not TFIIIB display changes in abundance in cellular extracts derived from these transitions.

These results support previous observations by Segil et al. [21], who affinity purified TFIID from G2/M-enriched cells and found by co-immunoprecipitation that TBP, TAF1, TAF4, TAF5, TAF7, TAF9, TAF10, TAF12 remained present in mitotic TFIID. Also, mitotic SL1 and the stable components of TFIIIB (TBP and BRF1) have been reported to remain intact [1,20,22,23]. We extend these analyses with a quantitative analysis of G2/M-specific TBP interactions of the recently identified SL1 subunit TAFI41/TAF1D [10], and of the TFIID subunits not analyzed so far, including TAF2, TAF3, TAF6, TAF8, TAF11.

### Cell cycle-induced phosphorylations on TAFs

**Figure 2:**
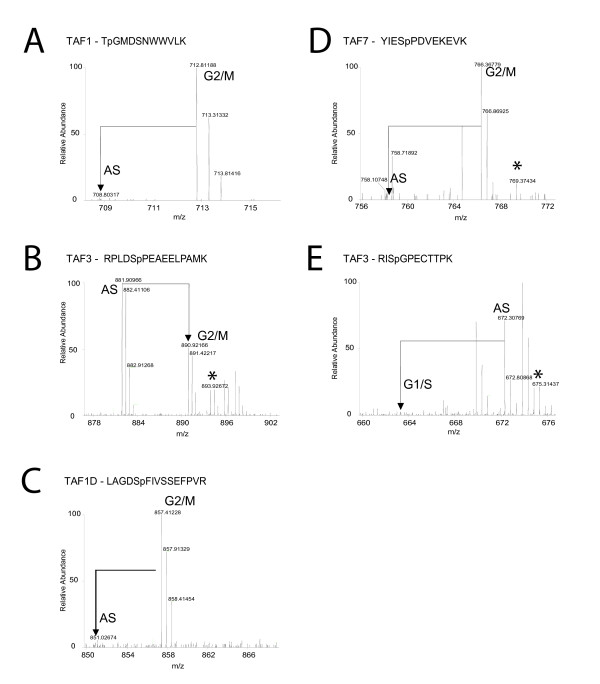
**Cell cycle-dependent phosphorylations on TAFs**. Quantitative mass spectra of highly cell cycle-regulated phosphorylations on TAFs. Peak pairs representing isotopic labeled phosphopeptides derived from asynchronous and cell cycle blocked cells are indicated. Peaks labeled * are derived from isotopic labeled arginine-to-proline conversion, and should be added to the 'heavy' peaks for quantification. Shown are G2/M-induced phosphorylations on TAF1 (A), TAF1D (C), and TAF7 (D); G1/S-induced dephosphorylation on TAF3 (E). (B) Cell cycle-independent phosphorylation on TAF3. All phosphorylations were quantified after correction for protein levels (see Table 1).

A number of novel phosphorylations on TAFs in the context of their protein complexes were detected and quantified (Fig 2, Table 1, and Additional Files 2 and 3). This revealed highly G2/M-specific phosphorylations of the TFIID subunits TAF1 (T876) and TAF7 (S159), and of the SL1 subunit TAF1D (S234) (Table 1, Fig 2a,d,c). The T876 phosphorylation of TAF1 was interesting because of its proximity to G716, which is mutated to aspartic acid in ts13 hamster cells resulting in a thermolabile form of TAF1 and to arrest of ts13 cells in G1 at 39.5°C [17]. We made use of these cells to test involvement of T876 in G1 progression. Both the phosphopreventing T876A and the phosphomimicking T876D mutation were similarly active as wild type TAF1 in this assay, suggesting that this residue is not essential for cell cycle progression (data not shown). We created ts13 cell lines that stably express the phosphomutants of TAF1 at similar levels compared to endogenous hamster TAF1. These cells displayed unchanged expression levels of a selected set of genes including the cell cycle-independent genes for cyclophilin B and glucose phosphate dehydrogenase, and the G2/M-specific genes for stk6 and cyclin B2 (data not shown). This suggested that phosphorylation of T876 is not critical for the cell cycle function of TAF1 or that it is redundant with other modifications. Slightly elevated (2.2-fold) phosphorylation of S543 TAF4 was detected in G2/M cells (Table 1). At G1/S, TAF3 was strongly dephosphorylated at S427, and to a lesser extend at S364 (Table 1, Fig 2e). Using a 2-fold cut off, other phosphorylations detected were cell cycle-independent. These included those on TAF3 (S183; Fig 2b), TAF6 (S532 and S653), and TAF1D (S40 and S137) (Table 1). All of these phosphorylations are novel, except S183 of TAF3, which appeared also cell cycle-independent in another study [31], and S653 of TAF6, which was not tested previously for cell cycle regulation [32].

**Table 1:** Quantification of phosphorylations on TBP interactors in cell cycle-blocked cells.

Protein	Peptide	Position	Phospopeptide ratio (G2/M:AS)	Protein ratio (G2/M:AS)	Phosphopeptide/Protein ratio (G2/M:AS)	Reference
TAF1	TpGMDSNWWVLK	T876	150.91	1.40	107.99	this study

TAF3	RPLDSpPEAEELPAMK	S183	1.38	1.60	0.86	this study; ref [31]

TAF4	SpPGVQPQLVLGGAA- QTASLGTATAVQTGTPQR	S543	3.13	1.42	2.20	this study

TAF7	YIESpPDVEKEVK	S159	15.77	1.36	11.58	this study

TAFI41/TAF1D	LAGDSpFIVSSEFPVR	S234	> 200	2.88	> 200	this study

						

**Protein**	**Peptide**	**Position**	**Phospopeptide ratio (G1/S:AS)**	**Protein ratio (G1/S:AS)**	**Phosphopeptide/Protein ratio (G1/S:AS)**	**Reference**

TAF3	RPLDSpPEAEELPAMK	S183	0.11	0.12	0.94	this study; ref [31]

TAF3	QIQTpPPDAGK	T364	0.05	0.12	0.39	this study

TAF3	RISpGPECTTPK	S427	0.02	0.12	0.19	this study

TAF6	AAAPPQPSpPPPTK	S532	0.07	0.11	0.62	this study

TAF6	QEAGDSpPPPAPGTPK	S653	0.12	0.11	1.13	this study; ref [32]

TAF7	YIESpPDVEKEVK	S159	0.11	0.17	0.63	this study

TAFI41/TAF1D	SRGSGFPFLESpENEK	S137	1.43	1.35	1.06	this study

TAFI41/TAF1D	TQCIPYSpPKGEK	S40	1.94	1.35	1.44	this study

Phosphorylations (indicated with a 'p' behind the modified residue) detected on TBP interactors were quantified in cell cycle-blocked cells relative to asynchronous cells and were corrected for protein levels. The sequence of the tryptic peptide detected is indicated.

A recent study using total proteome analysis also identified cell cycle-dependent phosphorylations of several TBP interactors including G1-specific phosphorylations on TAF7, and mitosis-specific phosphorylations on TAF7, TAF8, TAF9, and TAF1 [31]. Our analysis of TAFs in the context of their protein complexes can be distinguished from mapping of phosphosites in total cell lysates, which do not distinguish whether a certain TAF is present in complex with TBP or in another complex or as free protein. This is particularly relevant for TAF8 and TAF9, as they have been shown to be part of the Smal TAF and/or SAGA complexes [24,25]. We note that it is inherently difficult to determine the stoichiometry of a particular phosphorylation by mass spectrometry. This relates to potential differences in ionization properties of the phoshopeptide and its unmodified counterpart. For similar reasons it is not possible to exclude modification of a particular peptide.

**Figure 3:**
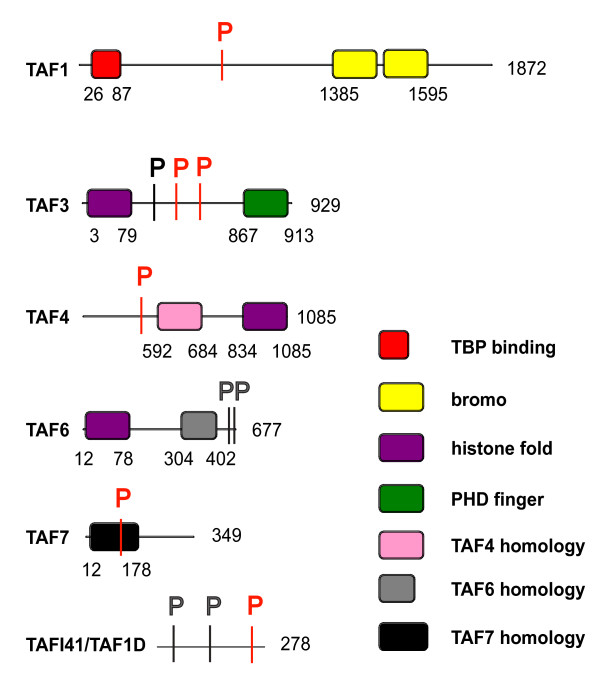
**Location of phosphorylations on TAFs with respect to protein domain organization**. Phosphosites are indicated with P using the following colour code: black, unchanged; red, cell cycle regulated (relative to asynchronous), using a 2-fold cut-off.

Interestingly, the phosphorylation at S427 on TAF3, which is downregulated at G1/S, is located in a region in which many other phosphosites have been mapped [33-35]. Out of all phosphosites detected in TAF3, all 17 (included the ones detected here) are located between S183 and T501 in the linker between the histone fold and PHD finger (Fig 3). Possibly the linker is involved in communication between the N-terminal histone fold, required for assembly into TFIID, and the C-terminal PHD finger, involved in recognition of histone H3 lysine 4 trimethyl-modified nucleosomes [36]. It is presently unknown whether any of these phosphorylations are also downregulated at G1/S, and whether this region is a true hotspot for phosphorylation or whether phosphorylations are just more easily detectable compared to other TAF3 regions.

The G2/M-specific phosphorylation at S159 of TAF7 is located in a TAF7-specific domain that spans residues 12-178 (Fig 3). Part of this domain overlaps with its TAF1-interaction domain, spanning residues 139-245. It is however unlikely that mitotic phosphorylation at S159 specifically affects the TAF7-TAF1 interaction, as both proteins have similar SILAC ratios in TBP purifications from G2/M cells (Fig 1c). TAF7 S159 lies just outside the domain that has been mapped to interact with transcriptional activators (spanning residues 38-118). This would be a way to inhibit the activity of TFIID at mitosis and silence transcription. Whether this possibility is true should be determined in future experiments.

### Bioinformatic analysis of phosphosites

**Figure 4:**
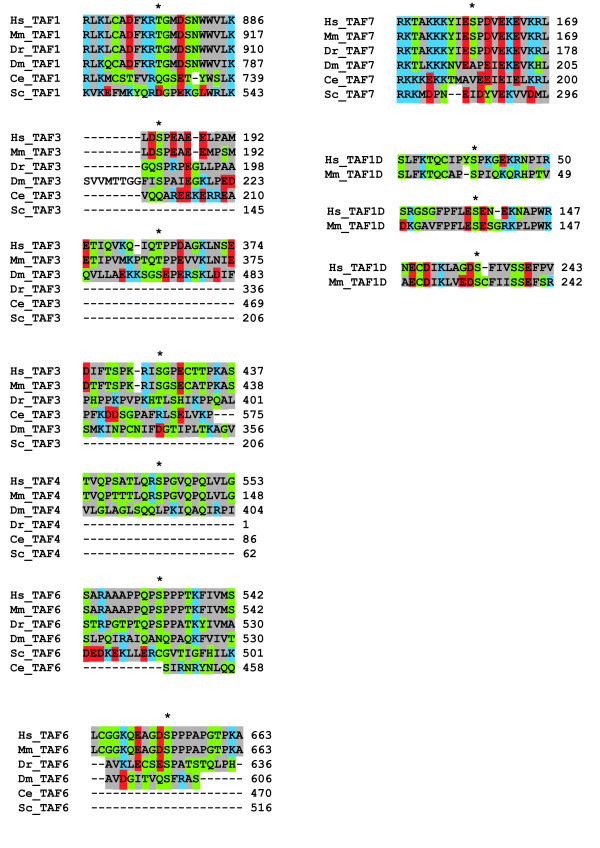
**Evolutionary conservation of phosphosites**. Aligments of sequences +/- 10 aa around the phosphosites between human (Hs), mouse (Mm), zebrafish (Dr), Drosophila (Dm), worm (Ce), and yeast (Sc), according to clustal W. Colouring was similar to clustal W: gray: small +hydrophobic AVFPMILW; red: acidic DE; blue: basic RK; green: neutral STYHCNGQ. * indicates the phosphorylated residue. UniProtKB/Swiss-Prot accession numbers [40] used are: Hs_TAF1: P21675; Mm_TAF1: Q80UV9; Dr_TAF1: Q1LYC2; Dm_TAF1: P51123; Ce_TAF1: Q9XUL9; Sc_TAF1: P46677; Hs_TAF3: Q5VWG9; Mm_TAF3: Q5HZG4; Dr_TAF3: Q5RH27; Dm_TAF3: Q9XZU7; Ce_TAF3: Q17907; Hs_TAF4: O00268; Mm_TAF4: A2AC70; Dm_TAF4: P47825; Hs_TAF6: P49848; Mm_TAF6: Q62311; Dr_TAF6: Q66HZ5; Dm_TAF6: P49847; Sc_TAF6: P53040; Hs_TAF7: Q15545; Mm_TAF7: Q9R1Co; Dr_TAF7: Q8JHG4; Hs_TAF1D: Q9H5J8; Mm_TAF1D: Q9D4V4.

Fig 3 shows the locations of the detected phosphorylations with respect to known protein domains. Most phosphorylations were present in linker regions between domains. To determine whether the phosphorylations were evolutionary conserved, an alignment of full length proteins from human, mouse, zebrafish, fruit flies, worm, and yeast was performed using clustal W [37] (Fig 4). TAFs present in TFIID were conserved in these organisms. However, the SL1-specific TAFI41/TAF1D was only conserved between human and mouse but not between human and lower eukaryotes. The alignments revealed that several residues found to be phosphorylated in human cells were conserved in mouse, zebrafish, and flies (TAF1 T876; TAF3 S183; TAF6 S653). Other residues were (semi) conserved (with either serine or threonine) from human to mouse and zebrafish (TAF3 S427; TAF6 S532, TAF7 S159), or from human to mouse and flies (TAF3 T364). Residues only conserved from human to mouse included TAF4 S543, and TAF1D S40, S137, and S234. None of the phosphorylated residues were conserved in worm or yeast, however, in some cases the aligned residue was negatively charged mimicking phosphorylation. This was observed for residues aligned with TAF1 T876 in yeast (D533) and with TAF3 S427 in flies (D346). Interestingly, some phosphorylations were present in regions that were highly conserved from human to yeast (TAF1 T876; TAF6 S532; TAF7 S159) or from human to flies or worm (TAF3 S18 and S427; TAF6 S653), suggesting that they may be involved in regulating important functions of these proteins.

## Conclusions

Recent advances in proteome technology allow a quantitative analysis of protein complexes and their post-translational modification status under various cellular conditions. Previous work suggested that TBP complexes play important roles in cell cycle progression, and that some of the TAFs remain associated with TBP and are subject to phosphorylation. Using SILAC and titanium dioxide enrichment of phosphopeptides, we here extend these analysis for all TAFs at the G2/M and G1/S transitions, and found that: 1)TBP complexes remain intact, 2) the abundances of TBP complexes in cellular cell extracts are cell cycle-dependent, and 3) numerous novel phosphorylation sites could be quantified. This indicated that subunits of TFIID and SL1 are subject to cell cycle-dependent phosphorylations. We speculate that phosphorylation of TAFs is involved in regulating the cell cycle dependent activity of TBP complexes.

## Methods

### Cell culture

Creation and culture of the HeLa S3 cell line stably expressing N-flag-HA-hTBP at near-endogenous levels has been described previously [26]. For SILAC analysis, cells were labelled using the heavy amino acids ^13^C_6_,^15^N_4_-arginine and ^13^C_6_,^15^N_2_-lysine or the light amino acids ^12^C_6_,^14^N_4_-arginine and ^12^C_6_,^14^N_2_-lysine as described [26]. Metabolic labeling was performed using heavy amino acids for G2/M cells compared to light amino acids for AS cells, and light amino acids for G1/S cells compared to heavy amino acids for AS cells. Scale-up of cell culture was performed using a 15 l bioreactor (Applikon, NL). Cells were blocked in G2/M using 60 ng/ml nocodazole for 17 hr. For cell cycle block at the G1/S transition, a double thymidine block was performed using 2.5 mM thymidine and a sequence of 18 hr for the first block, 8 hr release, and 18 hr for the second block. Cell cycle blocks were verified by FACS analysis of propidium iodide-stained cells.

### Preparation of protein extracts

Protein extracts were prepared based on Dignam et al. [30]. All handling was performed at 4°C. Cells were washed two times in PBS, and were allowed to swell in three packed cell volumes (PCVs) of buffer A (10 mM Tris-HCl pH 7.9, 20% glycerol, 1.5 mM MgCl_2_, 0.1 mM EDTA, 10 mM KCl, 1 mM DTT, 1% protease inhibitor cocktail (Sigma P8340), 1% phosphatase inhibitor cocktail 1 (Sigma P2850), 1% phosphatase inhibitor cocktail 2 (Sigma P5726). After centrifugation at 1500 rpm for 10 min, the cell pellet was resuspended in 3 PCV of buffer A. Cells were lysed by douncing using a tight pestle, and then centrifuged at 3000 rpm for 15 min. The supernatant was brought to 100 mM NaCl and represents the cytoplasmic extract (CE). The pellet was resuspended in 5 ml per 10^9 ^cells of buffer B (20 mM HEPES-NaOH pH 7.9, 25% glycerol, 1.5 mM MgCl_2_, 0.2 mM EDTA, 0.42 M NaCl, 1 mM DTT, protease and phosphatase inhibitors as above), and incubated for 30 min with rotation. This mixture was centrifuged at 15,000 rpm for 20 min. The supernatant represents the nuclear extract (NE).

### Affinity purification of TBP complexes

Extracts were dialyzed overnight in buffer C (20 mM HEPES-NaOH pH 7.9, 20% glycerol, 0.2 mM EDTA, 100 mM NaCl, 1 mM DTT, protease and phosphatase inhibitors as above). Extracts were centrifuged at 100,000 × g for 45 min to remove precipitated material. Extracts representing whole cell extract (CE + NE) were double affinity purified as described [26]. Extracts from asynchronous cells and cell cycle blocked cells were compared by SILAC and quantitative mass spectrometry. TBP complexes were purified separately to prevent exchange of subunits during the purification procedure as described [26]. Purified complexes were mixed at a 1:1 ratio based on the total protein content of the input material and denatured immediately by methanol/chloroform precipitation. Precipitated proteins were stored at -80°C until analysis by mass spectrometry.

### In-solution digestion of purified protein complexes

Purified protein complexes were dissolved in a solution of 8 M urea in 50 mM ammonium bicarbonate pH 8, and incubated with 5 μg of endoproteinase LysC (Roche Diagnostics) for 4 hr at 37°C. Following reduction and alkylation using 2 mM DTT and 4 mM iodoacetamide, respectively, the sample was diluted to 2 M urea with 50 mM ammonium bicarbonate pH 8, and incubated overnight with 5 μg trypsin at 37°C.

### SCX Chromatography and online TiO2 based two-dimensional chromatography

After digestion, the protein digests were desalted using a small plug of C18 material (3 M Empore C18 extraction disk) packed into a GELoader Tip similar to as previously described [38] onto which 10 μL of Aqua C18 (5 μm, 200 Å) material was placed. The eluate was dried completely. The G2/M sample was reconstituted in 20% (v/v) acetonitrile, 0.05% (v/v) formic acid and analyzed using strong cation exchange chromatography and subsequent fractions were analyzed on a TiO2 based 2D-nanoflow-HPLC as described [29]. The G1/S sample was reconstituted in 5% formic acid, and immediately analyzed on the TiO2 based 2D-nanoflow-HPLC system, without prior SCX fractionation as described by Pinkse et al. [29]

### Mass Spectrometry

The 2D-LC system was online coupled to a LTQ Orbitrap mass spectrometer (Thermo Electron, Bremen, Germany), which operated in data-dependent mode, automatically switching between MS, MS/MS, and neutral loss-driven MS^3 ^acquisition. Full-scan MS spectra (from *m*/*z *300 to 1500) were acquired in the Orbitrap with a resolution of 60,000 at *m*/*z *400 after accumulation to a target value of 500,000. The three most intense ions at a threshold above 5000 were selected for collision-induced fragmentation in the linear ion trap at a normalized collision energy of 35% after accumulation to a target value of 10,000. The data-dependent neutral loss settings were chosen to trigger an MS^3 ^event after a neutral loss of 49 ± 0.5 *m*/*z *units was detected in the most intense fragment ion.

### Protein Identification

In a post analysis process, all MS2 and MS3 spectra were converted to single DTA files using Bioworks software, Version 3.1. An in-house Perl-script was used to assign the original and accurate parent mass to all MS3 spectra, enabling accurate parent mass identification. For protein identification, MS/MS data were submitted to the International Protein Index (IPI) human (release 3.36; 69012 entries) using Mascot Version 2.2 (Matrix Science) with the following settings: 20 ppm and 0.8 Da deviation for precursor and fragment masses, respectively. Trypsin was specified as the proteolytic enzyme, and up to two missed cleavages were allowed. Carbamidomethyl cysteine was set as fixed modification; N-terminal acetylation, oxidized methionines, ^13^C_6_-^15^N_2 _lysine, ^13^C_6_-^15^N_4 _arginine and phosphorylation of serine and threonine residues were set as variable modifications. The following was done to verify identified phosphosites: 1) Mascot scores of potential phosphosites at alternative residues in the identified peptides were determined and were found to be lower compared to the phosphosites presented in all cases, 2) All MS/MS spectra were manually verified.

### Protein quantification

Relative quantification ratios of identified proteins were derived by MSQuant, which is open source software [39]. Briefly, peptide ratios between the monoisotopic peaks of "normal" and "heavy" forms of the peptide were calculated and averaged over consecutive MS cycles for the duration of their respective LC-MS peaks in the total ion chromatogram using FT-survey. Peptide ratios of the same protein were averaged to give protein abundance ratios as well as the respective standard deviation. Peptide ratios obtained by using the MSQuant software were all inspected manually. Our experiments, in agreement with data from other groups, showed that HeLa cells convert ^13^C_6_-^15^N_4_-arginine to ^13^C_5_-^15^N_1 _-proline. In these experiments the conversion was estimated as 13.5%. We corrected the peptide ratio for this conversion as described previously [26]. After the proline conversion correction, protein ratios were normalized on the TBP level.

### Bioinformatic analysis

Sequence aligments were made using clustal W software [37] using full cDNA sequences of TAFs and their homologs. The stretch containing the phosphorylated residue +/- 10 amino acids was manually edited. Omission of an alignment indicates that no homologous region or protein was identified.

## Competing interests

The authors declare that they have no competing interests.

## Authors' contributions

WWMP, AK, AJRH, and HTMT contributed to experimental design, data interpretation, and manuscript preparation. Mass spectrum analysis was performed by AK. Tissue culture, biochemical experiments, and bioinformatic analysis were performed by WWMP and MPAB. WWMP and AK contributed equally. AJRH and HTMT contributed equally. All authors read and approved the final manuscript.

## Supplementary Material

Additional file 1 Sequence coverage for identified TBP interactors. Click here for file

Additional file 2 MS/MS spectra and Mascot scores for identified phosphopeptides in the G2/M:AS sample. Click here for file

Additional file 3 MS/MS spectra and Mascot scores for identified phosphopeptides in the G1/S:AS sample. Click here for file
