# Supplementary material for: Quantitative mass spectrometry of TATA binding protein-containing complexes and subunit phosphorylations during the cell cycle
Source: Proteome Sci. 2009 Dec 24;7:46. doi: 10.1186/1477-5956-7-46 (PMC2804597; doi:10.1186/1477-5956-7-46)
Supplement: Additional file 2 — MS/MS spectra and Mascot scores for identified phosphopeptides in the G2/M:AS sample. [file 1477-5956-7-46-S2.PDF]

TAF1; TpGMDSNWWVLK + 13C6-15N2 (K);
Phospho (T) Ion score: 64

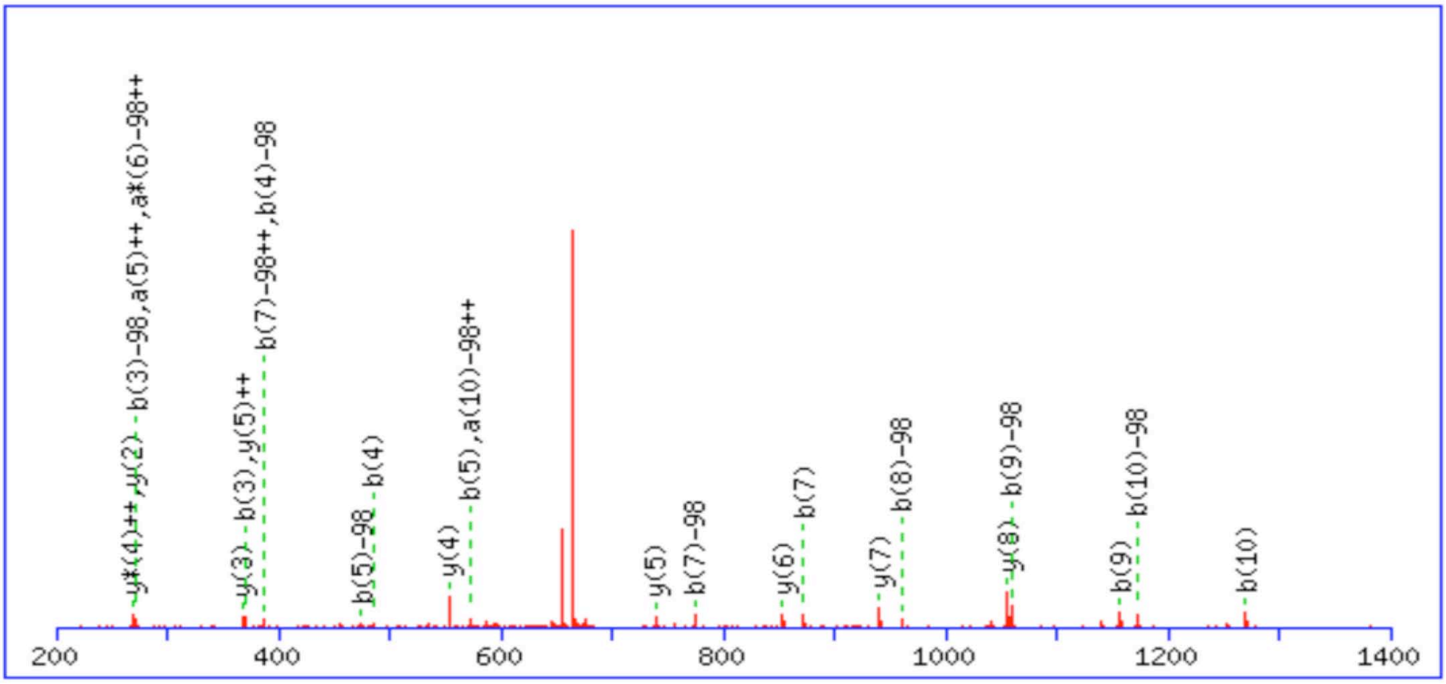

#	a	a++	a*	a*++	b	b++	b*	b*++	Seq.	y	y++	y*	y*++	#
1	56.0495	28.5284			84.0444	42.5258			T					11
2	113.0709	57.0391			141.0658	71.0366			G	1243.6020	622.3046	1226.5754	613.7913	10
3	244.1114	122.5593			**272.1063**	136.5568			M	1186.5805	593.7939	1169.5539	585.2806	9
4	359.1384	180.0728			**387.1333**	194.0703			D	**1055.5400**	528.2736	1038.5135	519.7604	8
5	446.1704	223.5888			**474.1653**	237.5863			S	**940.5131**	470.7602	923.4865	462.2469	7
6	560.2133	280.6103	543.1868	**272.0970**	588.2082	294.6078	571.1817	286.0945	N	**853.4810**	427.2442	836.4545	418.7309	6
7	746.2926	373.6500	729.2661	365.1367	**774.2875**	**387.6474**	757.2610	379.1341	W	**739.4381**	**370.2227**	722.4116	361.7094	5
8	932.3719	466.6896	915.3454	458.1763	**960.3669**	480.6871	943.3403	472.1738	W	**553.3588**	277.1830	536.3323	**268.6698**	4
9	1031.4404	516.2238	1014.4138	507.7105	**1059.4353**	530.2213	1042.4087	521.7080	V	**367.2795**	184.1434	350.2529	175.6301	3
10	1144.5244	**572.7658**	1127.4979	564.2526	**1172.5193**	586.7633	1155.4928	578.2500	L	**268.2111**	134.6092	251.1845	126.0959	2
11									K	155.1270	78.0671	138.1005	69.5539	1

TAF3; RPLDSpPEAEELPAMK +
Phospho (S) Ion score: 62

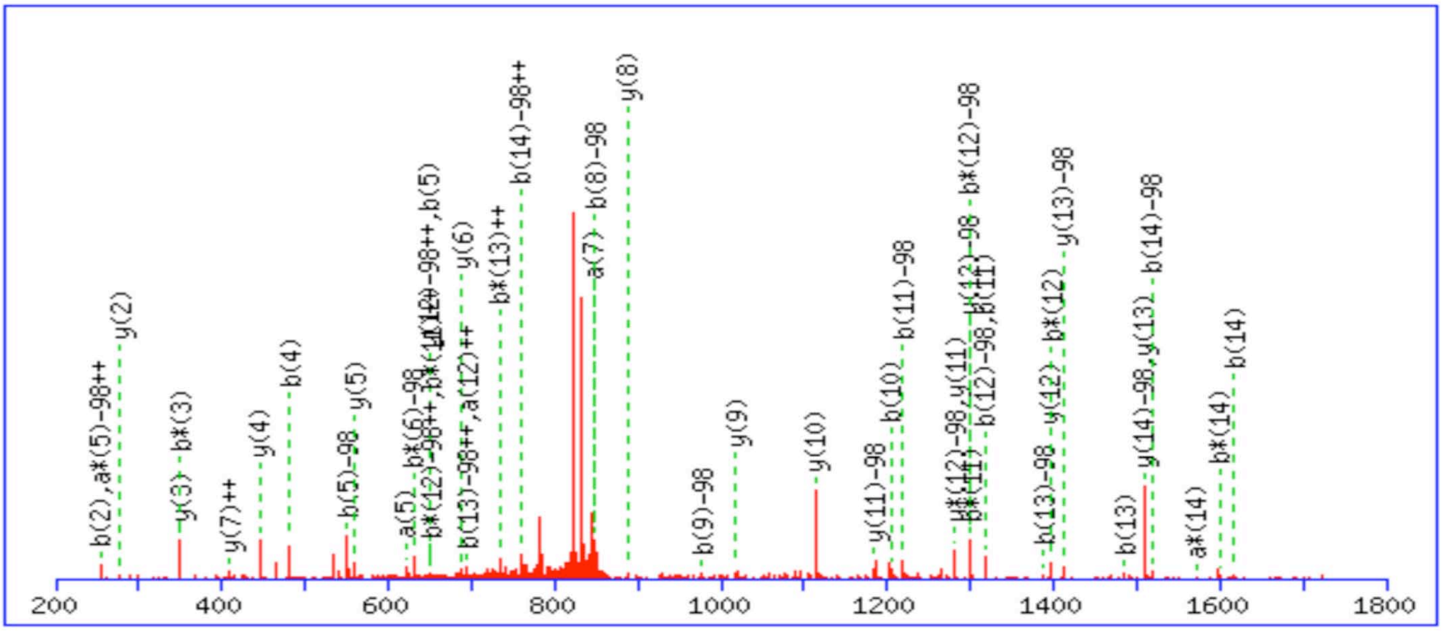

#	a	a++	a*	a*++	b	b++	b*	b*++	Seq.	y	y++	y*	y*++	#
1	129.1135	65.0604	112.0869	56.5471	157.1084	79.0578	140.0818	70.5446	R					15
2	226.1662	113.5868	209.1397	105.0735	**254.1612**	127.5842	237.1346	119.0709	P	**1508.7301**	754.8687	1491.7036	746.3554	14
3	339.2503	170.1288	322.2238	161.6155	367.2452	184.1262	**350.2187**	175.6130	L	**1411.6774**	706.3423	1394.6508	697.8290	13
4	454.2772	227.6423	437.2507	219.1290	**482.2722**	241.6397	465.2456	233.1264	D	**1298.5933**	**649.8003**	**1281.5667**	641.2870	12
5	523.2987	262.1530	506.2722	**253.6397**	**551.2936**	276.1504	534.2671	267.6372	S	**1183.5663**	592.2868	1166.5398	583.7735	11
6	620.3515	310.6794	603.3249	302.1661	648.3464	324.6768	**631.3198**	316.1636	P	**1114.5449**	557.7761	1097.5183	549.2628	10
7	749.3941	375.2007	732.3675	366.6874	777.3890	389.1981	760.3624	380.6848	E	**1017.4921**	509.2497	1000.4656	500.7364	9
8	820.4312	410.7192	803.4046	402.2059	**848.4261**	424.7167	831.3995	416.2034	A	**888.4495**	444.7284	871.4230	436.2151	8
9	949.4738	475.2405	932.4472	466.7272	**977.4687**	489.2380	960.4421	480.7247	E	817.4124	**409.2098**	800.3859	400.6966	7
10	1078.5164	539.7618	1061.4898	531.2485	1106.5113	553.7593	1089.4847	545.2460	E	**688.3698**	344.6886	671.3433	336.1753	6
11	1191.6004	596.3038	1174.5739	587.7906	**1219.5953**	610.3013	1202.5688	601.7880	L	**559.3272**	280.1673	542.3007	271.6540	5
12	1288.6532	644.8302	1271.6266	636.3170	**1316.6481**	658.8277	**1299.6216**	**650.3144**	P	**446.2432**	223.6252	429.2166	215.1119	4
13	1359.6903	680.3488	1342.6637	671.8355	**1387.6852**	**694.3462**	1370.6587	685.8330	A	**349.1904**	175.0988	332.1639	166.5856	3
14	1490.7308	745.8690	1473.7042	737.3558	**1518.7257**	**759.8665**	1501.6991	751.3532	M	**278.1533**	139.5803	261.1267	131.0670	2
15									K	147.1128	74.0600	130.0863	65.5468	1

TAF4; SpPGVQPQLVLGGAAQTASLGTATAVQTGTPQR + 13C6-15N4 (R); Phospho (S) Ion score: 52

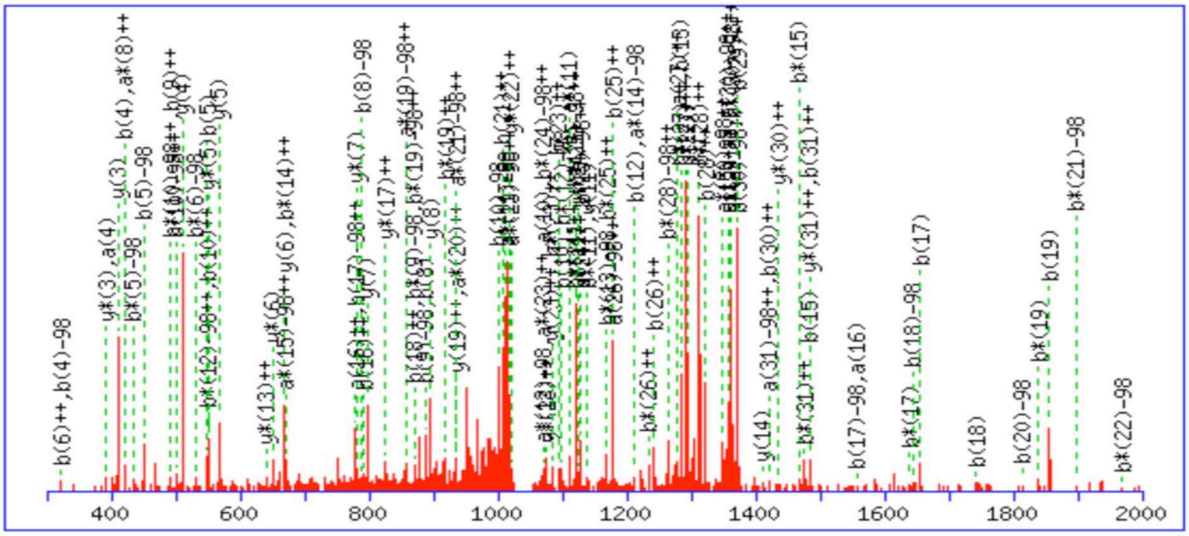

#	a	a++	a*	a*++	b	b++	b*	b*++	Seq.	y	y++	y*	y*++	#
1	140.0107	70.5090			168.0056	84.5065			S					32
2	237.0635	119.0354			265.0584	133.0328			P	2985.5992	1493.3032	2968.5726	1484.7899	31
3	294.0849	147.5461			322.0799	161.5436			G	2888.5464	1444.7768	2871.5198	1436.2636	30
4	393.1534	197.0803			421.1483	211.0778			V	2831.5249	1416.2661	2814.4984	1407.7528	29
5	521.2119	261.1096	504.1854	252.5963	549.2068	275.1071	532.1803	266.5938	Q	2732.4565	1366.7319	2715.4300	1358.2186	28
6	618.2647	309.6360	601.2381	301.1227	646.2596	323.6334	629.2331	315.1202	P	2604.3979	1302.7026	2587.3714	1294.1893	27
7	746.3233	373.6653	729.2967	365.1520	774.3182	387.6627	757.2916	379.1495	Q	2507.3452	1254.1762	2490.3186	1245.6629	26
8	859.4073	430.2073	842.3808	421.6940	887.4023	444.2048	870.3757	435.6915	L	2379.2866	1190.1469	2362.2600	1181.6337	25
9	958.4758	479.7415	941.4492	471.2282	986.4707	493.7390	969.4441	485.2257	V	2266.2025	1133.6049	2249.1760	1125.0916	24
10	1071.5598	536.2835	1054.5333	527.7703	1099.5547	550.2810	1082.5282	541.7677	L	2167.1341	1084.0707	2150.1076	1075.5574	23
11	1128.5813	564.7943	1111.5547	556.2810	1156.5762	578.7917	1139.5496	570.2785	G	2054.0500	1027.5287	2037.0235	1019.0154	22
12	1185.6027	593.3050	1168.5762	584.7917	1213.5977	607.3025	1196.5711	598.7892	G	1997.0286	999.0179	1980.0020	990.5047	21
13	1256.6399	628.8236	1239.6133	620.3103	1284.6348	642.8210	1267.6082	634.3078	A	1940.0071	970.5072	1922.9806	961.9939	20
14	1327.6770	664.3421	1310.6504	655.8288	1355.6719	678.3396	1338.6453	669.8263	A	1868.9700	934.9886	1851.9435	926.4754	19
15	1455.7356	728.3714	1438.7090	719.8581	1483.7305	742.3689	1466.7039	733.8556	Q	1797.9329	899.4701	1780.9063	890.9568	18
16	1556.7832	778.8953	1539.7567	770.3820	1584.7781	792.8927	1567.7516	784.3794	T	1669.8743	835.4408	1652.8478	826.9275	17
17	1627.8203	814.4138	1610.7938	805.9005	1655.8153	828.4113	1638.7887	819.8980	A	1568.8266	784.9170	1551.8001	776.4037	16
18	1714.8524	857.9298	1697.8258	849.4165	1742.8473	871.9273	1725.8207	863.4140	S	1497.7895	749.3984	1480.7630	740.8851	15
19	1827.9364	914.4719	1810.9099	905.9586	1855.9314	928.4693	1838.9048	919.9560	L	1410.7575	705.8824	1393.7309	697.3691	14
20	1884.9579	942.9826	1867.9314	934.4693	1912.9528	956.9800	1895.9263	948.4668	G	1297.6734	649.3404	1280.6469	640.8271	13
21	1986.0056	993.5064	1968.9790	984.9932	2014.0005	1007.5039	1996.9739	998.9906	T	1240.6520	620.8296	1223.6254	612.3163	12
22	2057.0427	1029.0250	2040.0161	1020.5117	2085.0376	1043.0224	2068.0111	1034.5092	A	1139.6043	570.3058	1122.5777	561.7925	11
23	2158.0904	1079.5488	2141.0638	1071.0355	2186.0853	1093.5463	2169.0587	1085.0330	T	1068.5672	534.7872	1051.5406	526.2739	10
24	2229.1275	1115.0674	2212.1009	1106.5541	2257.1224	1129.0648	2240.0959	1120.5516	A	967.5195	484.2634	950.4929	475.7501	9
25	2328.1959	1164.6016	2311.1694	1156.0883	2356.1908	1178.5990	2339.1643	1170.0858	V	896.4824	448.7448	879.4558	440.2316	8
26	2456.2545	1228.6309	2439.2279	1220.1176	2484.2494	1242.6283	2467.2228	1234.1151	Q	797.4140	399.2106	780.3874	390.6973	7
27	2557.3022	1279.1547	2540.2756	1270.6414	2585.2971	1293.1522	2568.2705	1284.6389	T	669.3554	335.1813	652.3288	326.6681	6
28	2614.3236	1307.6654	2597.2971	1299.1522	2642.3185	1321.6629	2625.2920	1313.1496	G	568.3077	284.6575	551.2812	276.1442	5
29	2715.3713	1358.1893	2698.3448	1349.6760	2743.3662	1372.1867	2726.3397	1363.6735	T	511.2862	256.1468	494.2597	247.6335	4
30	2812.4241	1406.7157	2795.3975	1398.2024	2840.4190	1420.7131	2823.3924	1412.1999	P	410.2386	205.6229	393.2120	197.1096	3
31	2940.4826	1470.7450	2923.4561	1462.2317	2968.4776	1484.7424	2951.4510	1476.2291	Q	313.1858	157.0965	296.1593	148.5833	2
32									R	185.1272	93.0672	168.1007	84.5540	1

TAF7; YIESpPDVEKEVK + 2 13C6-15N2 (K); Phospho (S) Ion score: 49

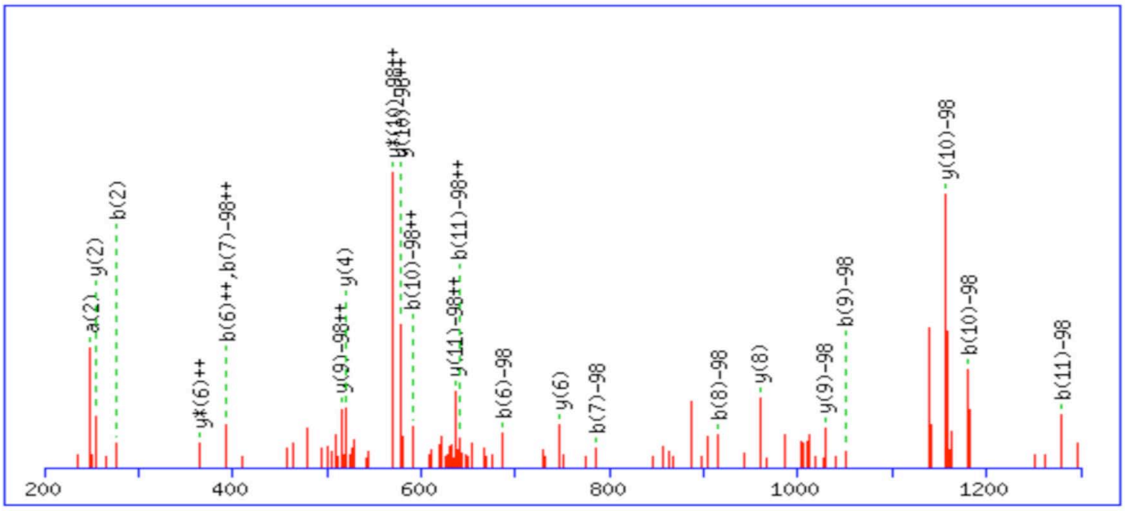

#	a	a++	a*	a*++	b	b++	b*	b*++	Seq.	y	y++	y*	y*++	#
1	136.0757	68.5415			164.0706	82.5389			Y					12
2	**249.1598**	125.0835			**277.1547**	139.0810			I	1270.6860	**635.8466**	1253.6595	627.3334	11
3	378.2023	189.6048			406.1973	203.6023			E	**1157.6019**	**579.3046**	1140.5754	**570.7913**	10
4	447.2238	224.1155			475.2187	238.1130			S	**1028.5594**	**514.7833**	1011.5328	506.2700	9
5	544.2766	272.6419			572.2715	286.6394			P	**959.5379**	480.2726	942.5114	471.7593	8
6	659.3035	330.1554			**687.2984**	344.1529			D	862.4851	431.7462	845.4586	423.2329	7
7	758.3719	379.6896			**786.3668**	**393.6871**			V	**747.4582**	374.2327	730.4316	**365.7195**	6
8	887.4145	444.2109			**915.4094**	458.2084			E	648.3898	324.6985	631.3632	316.1853	5
9	1023.5237	512.2655	1006.4971	503.7522	**1051.5186**	526.2629	1034.4921	517.7497	K	**519.3472**	260.1772	502.3206	251.6640	4
10	1152.5663	576.7868	1135.5397	568.2735	**1180.5612**	**590.7842**	1163.5346	582.2710	E	383.2380	192.1226	366.2115	183.6094	3
11	1251.6347	626.3210	1234.6081	617.8077	**1279.6296**	**640.3184**	1262.6031	631.8052	V	**254.1954**	127.6014	237.1689	119.0881	2
12									K	155.1270	78.0671	138.1005	69.5539	1

TAF1D; LAGDSpFIVSSEFPVR + 13C6-15N4 (R); Phospho (S) Ion score: 59

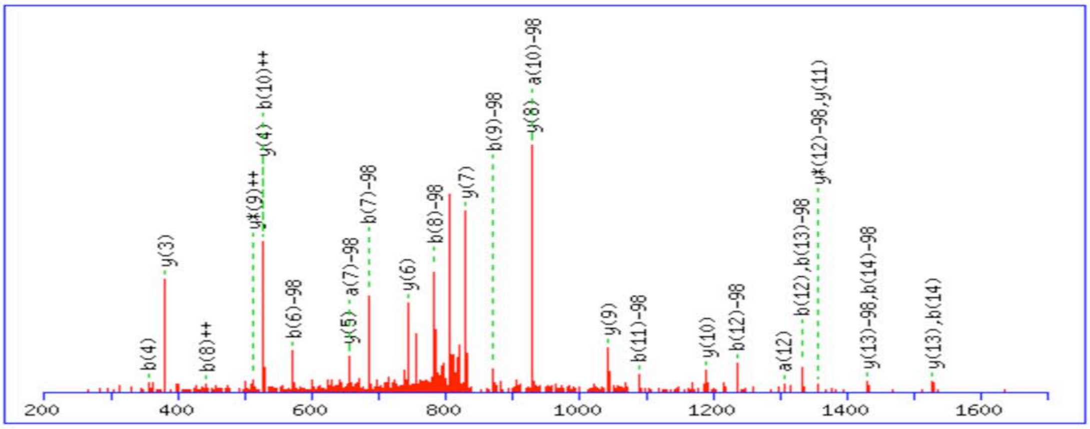

#	a	a++	b	b++	Seq.	y	y++	y*	y*++	#
1	86.0964	43.5519	114.0913	57.5493	L					15
2	157.1335	79.0704	185.1285	93.0679	A	1502.7513	751.8793	1485.7248	743.3660	14
3	214.1550	107.5811	242.1499	121.5786	G	**1431.7142**	716.3607	1414.6877	707.8475	13
4	329.1819	165.0946	**357.1769**	179.0921	D	1374.6928	687.8500	**1357.6662**	679.3367	12
5	398.2034	199.6053	426.1983	213.6028	S	1259.6658	630.3365	1242.6393	621.8233	11
6	545.2718	273.1395	**573.2667**	287.1370	F	**1190.6444**	595.8258	1173.6178	587.3125	10
7	**658.3559**	329.6816	**686.3508**	343.6790	I	**1043.5759**	522.2916	1026.5494	**513.7783**	9
8	757.4243	379.2158	**785.4192**	393.2132	V	**930.4919**	465.7496	913.4653	457.2363	8
9	844.4563	422.7318	**872.4512**	436.7293	S	**831.4235**	416.2154	814.3969	407.7021	7
10	**931.4883**	466.2478	959.4833	480.2453	S	**744.3914**	372.6994	727.3649	364.1861	6
11	1060.5309	530.7691	**1088.5259**	544.7666	E	**657.3594**	329.1833	640.3329	320.6701	5
12	1207.5994	604.3033	**1235.5943**	618.3008	F	**528.3168**	264.6620	511.2903	256.1488	4
13	1304.6521	652.8297	**1332.6470**	666.8272	P	**381.2484**	191.1278	364.2219	182.6146	3
14	1403.7205	702.3639	**1431.7155**	716.3614	V	284.1956	142.6015	267.1691	134.0882	2
15					R	185.1272	93.0672	168.1007	84.5540	1
