# Supplementary material for: Quantitative mass spectrometry of TATA binding protein-containing complexes and subunit phosphorylations during the cell cycle
Source: Proteome Sci. 2009 Dec 24;7:46. doi: 10.1186/1477-5956-7-46 (PMC2804597; doi:10.1186/1477-5956-7-46)
Supplement: Additional file 3 — MS/MS spectra and Mascot scores for identified phosphopeptides in the G1/S:AS sample. [file 1477-5956-7-46-S3.PDF]

TAF3; RPLDSpPEAEELPAMK + Phospho (S)
Ion score: 31

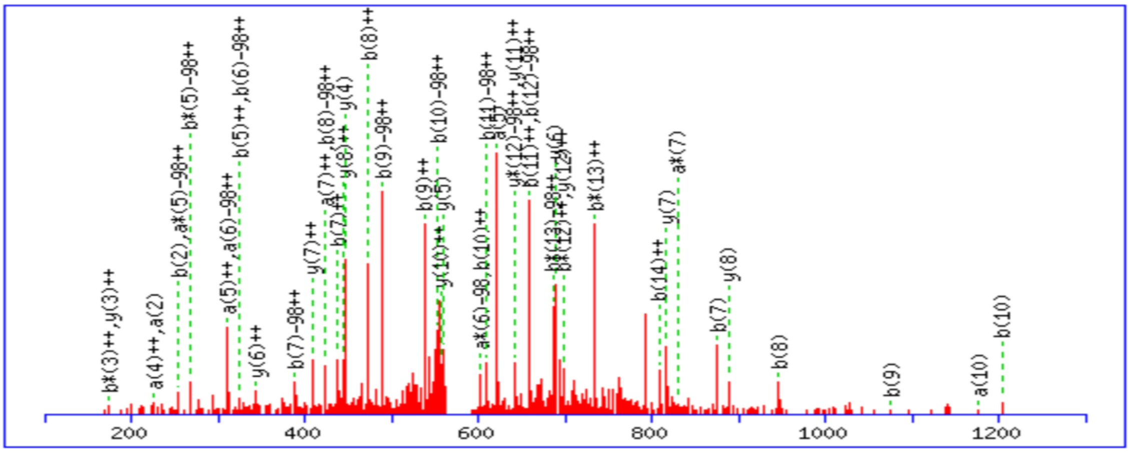

#	a	a++	a*	a*++	b	b++	b*	b*++	Seq.	y	y++	y*	y*++	#
1	129.1135	65.0604	112.0869	56.5471	157.1084	79.0578	140.0818	70.5446	R					15
2	**226.1662**	113.5868	209.1397	105.0735	**254.1612**	127.5842	237.1346	119.0709	P	1606.7070	803.8571	1589.6805	795.3439	14
3	339.2503	170.1288	322.2238	161.6155	367.2452	184.1262	350.2187	**175.6130**	L	1509.6542	755.3308	1492.6277	746.8175	13
4	454.2772	**227.6423**	437.2507	219.1290	482.2722	241.6397	465.2456	233.1264	D	1396.5702	**698.7887**	1379.5436	690.2755	12
5	**621.2756**	**311.1414**	604.2490	302.6282	649.2705	**325.1389**	632.2440	316.6256	S	1281.5432	**641.2753**	1264.5167	632.7620	11
6	718.3284	359.6678	701.3018	351.1545	746.3233	373.6653	729.2967	365.1520	P	1114.5449	**557.7761**	1097.5183	549.2628	10
7	847.3710	**424.1891**	**830.3444**	415.6758	**875.3659**	**438.1866**	858.3393	429.6733	E	1017.4921	509.2497	1000.4656	500.7364	9
8	918.4081	459.7077	901.3815	451.1944	**946.4030**	**473.7051**	929.3764	465.1919	A	**888.4495**	**444.7284**	871.4230	436.2151	8
9	1047.4507	524.2290	1030.4241	515.7157	**1075.4456**	**538.2264**	1058.4190	529.7132	E	**817.4124**	**409.2098**	800.3859	400.6966	7
10	**1176.4933**	588.7503	1159.4667	580.2370	**1204.4882**	**602.7477**	1187.4616	594.2344	E	**688.3698**	**344.6886**	671.3433	336.1753	6
11	1289.5773	645.2923	1272.5508	636.7790	1317.5722	**659.2898**	1300.5457	650.7765	L	**559.3272**	280.1673	542.3007	271.6540	5
12	1386.6301	693.8187	1369.6035	685.3054	1414.6250	707.8161	1397.5984	**699.3029**	P	**446.2432**	223.6252	429.2166	215.1119	4
13	1457.6672	729.3372	1440.6406	720.8240	1485.6621	743.3347	1468.6356	**734.8214**	A	349.1904	**175.0988**	332.1639	166.5856	3
14	1588.7077	794.8575	1571.6811	786.3442	1616.7026	**808.8549**	1599.6760	800.3417	M	278.1533	139.5803	261.1267	131.0670	2
15									K	147.1128	74.0600	130.0863	65.5468	1

TAF3; QIQTpPPDAGK + Phospho (T)
Ion score: 25

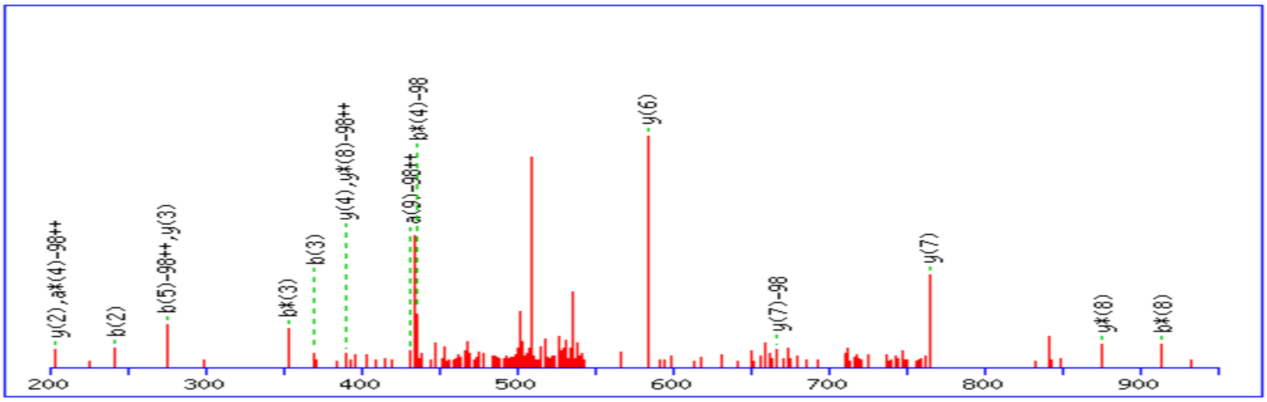

#	a	a^{++}	a*	a^{*++}	b	b^{++}	b*	b^{*++}	Seq.	y	y^{++}	y*	y^{*++}	#
1	101.0709	51.0391	84.0444	42.5258	129.0659	65.0366	112.0393	56.5233	Q					10
2	214.1550	107.5811	197.1285	99.0679	**242.1499**	121.5786	225.1234	113.0653	I	908.4836	454.7454	891.4571	446.2322	9
3	342.2136	171.6104	325.1870	163.0972	**370.2085**	185.6079	**353.1819**	177.0946	Q	795.3995	398.2034	778.3730	**389.6901**	8
4	425.2507	213.1290	408.2241	**204.6157**	453.2456	227.1264	**436.2191**	218.6132	T	**667.3410**	334.1741	650.3144	325.6608	7
5	522.3035	261.6554	505.2769	253.1421	550.2984	**275.6528**	533.2718	267.1395	P	**584.3039**	292.6556	567.2773	284.1423	6
6	619.3562	310.1817	602.3297	301.6685	647.3511	324.1792	630.3246	315.6659	P	487.2511	244.1292	470.2245	235.6159	5
7	734.3832	367.6952	717.3566	359.1819	762.3781	381.6927	745.3515	373.1794	D	**390.1983**	195.6028	373.1718	187.0895	4
8	805.4203	403.2138	788.3937	394.7005	833.4152	417.2112	816.3886	408.6980	A	**275.1714**	138.0893	258.1448	129.5761	3
9	862.4417	**431.7245**	845.4152	423.2112	890.4367	445.7220	873.4101	437.2087	G	**204.1343**	102.5708	187.1077	94.0575	2
10									K	147.1128	74.0600	130.0863	65.5468	1

TAF3; RISpGPECTTPK + Label:13C(6)15N(2) (K); Label:13C(6)15N(4) (R); Phospho (S)
Ion score: 17

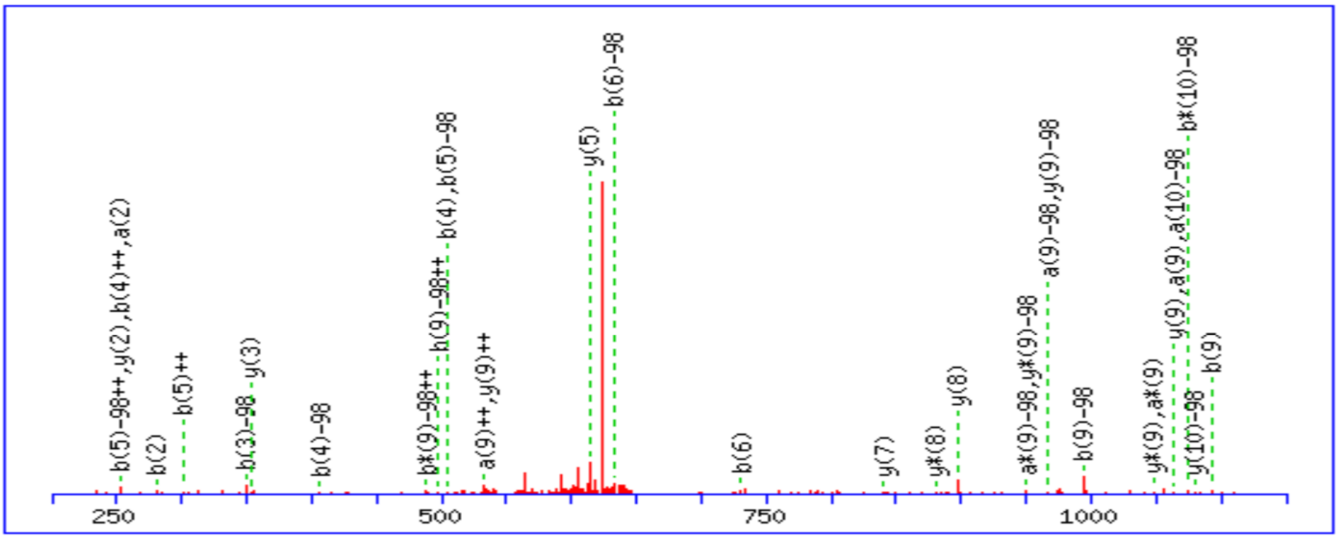

#	a	a++	a*	a*++	b	b++	b*	b*++	Seq.	y	y++	y*	y*++	#
1	139.1217	70.0645	122.0952	61.5512	167.1167	84.0620	150.0901	75.5487	R					11
2	**252.2058**	126.6065	235.1793	118.0933	**280.2007**	140.6040	263.1742	132.0907	I	**1079.5281**	540.2677	1062.5016	531.7544	10
3	321.2273	161.1173	304.2007	152.6040	**349.2222**	175.1147	332.1956	166.6015	S	**966.4441**	483.7257	**949.4175**	475.2124	9
4	378.2487	189.6280	361.2222	181.1147	**406.2436**	203.6255	389.2171	195.1122	G	**897.4226**	449.2149	**880.3961**	440.7017	8
5	475.3015	238.1544	458.2749	229.6411	**503.2964**	**252.1518**	486.2699	243.6386	P	**840.4011**	420.7042	823.3746	412.1909	7
6	604.3441	302.6757	587.3175	294.1624	**632.3390**	316.6731	615.3124	308.1599	E	743.3484	372.1778	726.3218	363.6646	6
7	764.3747	382.6910	747.3482	374.1777	792.3696	396.6885	775.3431	388.1752	C	**614.3058**	307.6565	597.2792	299.1433	5
8	865.4224	433.2148	848.3959	424.7016	893.4173	447.2123	876.3908	438.6990	T	454.2751	227.6412	437.2486	219.1279	4
9	**966.4701**	483.7387	**949.4435**	475.2254	**994.4650**	**497.7361**	977.4385	**489.2229**	T	**353.2275**	177.1174	336.2009	168.6041	3
10	**1063.5229**	532.2651	1046.4963	523.7518	1091.5178	546.2625	**1074.4912**	537.7492	P	**252.1798**	126.5935	235.1532	118.0803	2
11									K	155.1270	78.0671	138.1005	69.5539	1

Additional File 3

TAF6; AAAPPQPSpPPPTK + Label:13C(6)15N(2) (K); Phospho (S)
Ion score: 25

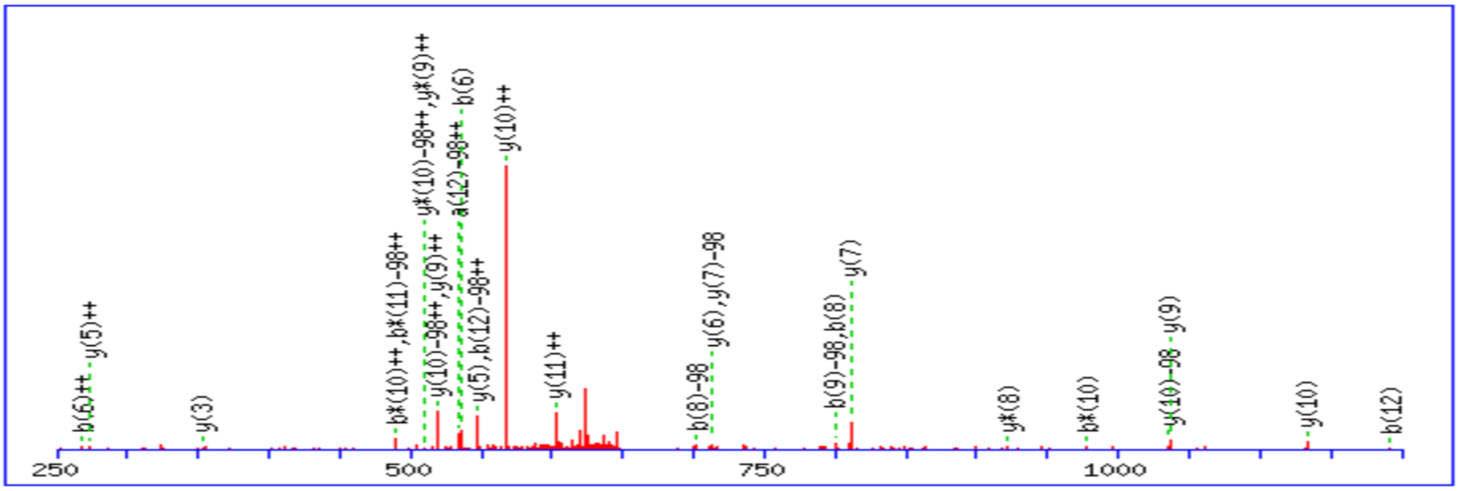

#	a	a++	a*	a*++	b	b++	b*	b*++	Seq.	y	y++	y*	y*++	#
1	44.0495	22.5284			72.0444	36.5258			A					13
2	115.0866	58.0469			143.0815	72.0444			A	1275.6224	638.3149	1258.5959	629.8016	12
3	186.1237	93.5655			214.1186	107.5629			A	1204.5853	**602.7963**	1187.5588	594.2830	11
4	283.1765	142.0919			311.1714	156.0893			P	**1133.5482**	**567.2777**	1116.5217	558.7645	10
5	380.2292	190.6183			408.2241	204.6157			P	**1036.4954**	**518.7514**	1019.4689	**510.2381**	9
6	508.2878	254.6475	491.2613	246.1343	**536.2827**	**268.6450**	519.2562	260.1317	Q	939.4427	470.2250	**922.4161**	461.7117	8
7	605.3406	303.1739	588.3140	294.6607	633.3355	317.1714	616.3089	308.6581	P	**811.3841**	406.1957	794.3575	397.6824	7
8	772.3389	386.6731	755.3124	378.1598	**800.3338**	400.6706	783.3073	392.1573	S	**714.3313**	357.6693	697.3048	349.1560	6
9	869.3917	435.1995	852.3651	426.6862	897.3866	449.1969	880.3601	440.6837	P	**547.3330**	**274.1701**	530.3064	265.6569	5
10	966.4445	483.7259	949.4179	475.2126	994.4394	497.7233	**977.4128**	**489.2100**	P	450.2802	225.6437	433.2537	217.1305	4
11	1063.4972	532.2522	1046.4707	523.7390	1091.4921	546.2497	1074.4656	537.7364	P	**353.2275**	177.1174	336.2009	168.6041	3
12	1164.5449	582.7761	1147.5183	574.2628	**1192.5398**	596.7735	1175.5133	588.2603	T	256.1747	128.5910	239.1481	120.0777	2
13									K	155.1270	78.0671	138.1005	69.5539	1

TAF6; QEAGDSpPPPAPGTPK + Phospho (S)
Ion score: 26

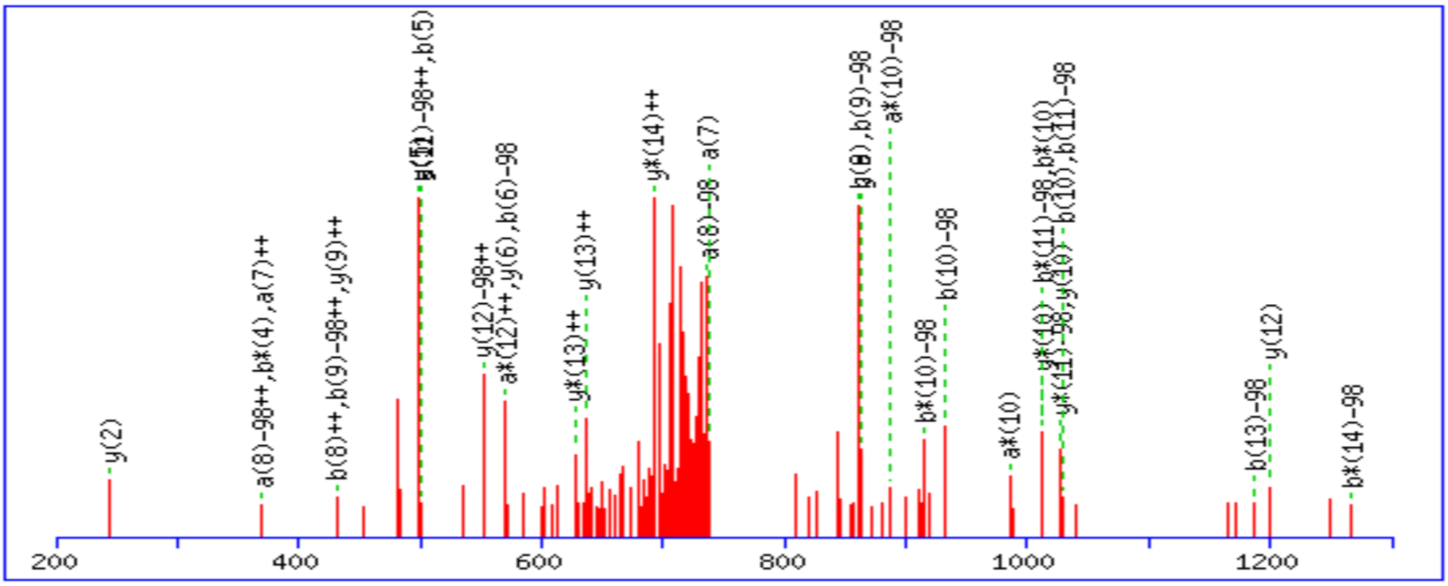

#	a	a^{++}	a*	a^{*++}	b	b^{++}	b*	b^{*++}	Seq.	y	y^{++}	y*	y^{*++}	#
1	101.0709	51.0391	84.0444	42.5258	129.0659	65.0366	112.0393	56.5233	Q					15
2	230.1135	115.5604	213.0870	107.0471	258.1084	129.5579	241.0819	121.0446	E	1302.6325	651.8199	1285.6059	643.3066	14
3	301.1506	151.0790	284.1241	142.5657	329.1456	165.0764	312.1190	156.5631	A	1173.5899	587.2986	1156.5633	578.7853	13
4	358.1721	179.5897	341.1456	171.0764	386.1670	193.5872	**369.1405**	185.0739	G	1102.5527	**551.7800**	1085.5262	543.2667	12
5	473.1991	237.1032	456.1725	228.5899	**501.1940**	251.1006	484.1674	242.5873	D	1045.5313	523.2693	**1028.5047**	514.7560	11
6	542.2205	271.6139	525.1940	263.1006	**570.2154**	285.6114	553.1889	277.0981	S	930.5043	465.7558	913.4778	457.2425	10
7	639.2733	320.1403	622.2467	311.6270	667.2682	334.1377	650.2416	325.6245	P	**861.4829**	**431.2451**	844.4563	422.7318	9
8	**736.3260**	**368.6667**	719.2995	360.1534	764.3210	382.6641	747.2944	374.1508	P	764.4301	382.7187	747.4036	374.2054	8
9	833.3788	417.1930	816.3523	408.6798	**861.3737**	**431.1905**	844.3472	422.6772	P	667.3774	334.1923	650.3508	325.6790	7
10	904.4159	452.7116	**887.3894**	444.1983	**932.4108**	466.7091	**915.3843**	458.1958	A	**570.3246**	285.6659	553.2980	277.1527	6
11	1001.4687	**501.2380**	984.4421	492.7247	**1029.4636**	515.2354	**1012.4370**	506.7222	P	**499.2875**	250.1474	482.2609	241.6341	5
12	1058.4901	529.7487	1041.4636	521.2354	1086.4851	543.7462	1069.4585	535.2329	G	402.2347	201.6210	385.2082	193.1077	4
13	1159.5378	580.2725	1142.5113	571.7593	**1187.5327**	594.2700	1170.5062	585.7567	T	345.2132	173.1103	328.1867	164.5970	3
14	1256.5906	628.7989	1239.5640	620.2857	1284.5855	642.7964	**1267.5590**	634.2831	P	**244.1656**	122.5864	227.1390	114.0731	2
15									K	147.1128	74.0600	130.0863	65.5468	1

TAF7; YIESpPDVEKEVK + 2 Label:13C(6)15N(2) (K); Phospho (S)
Ion score: 35

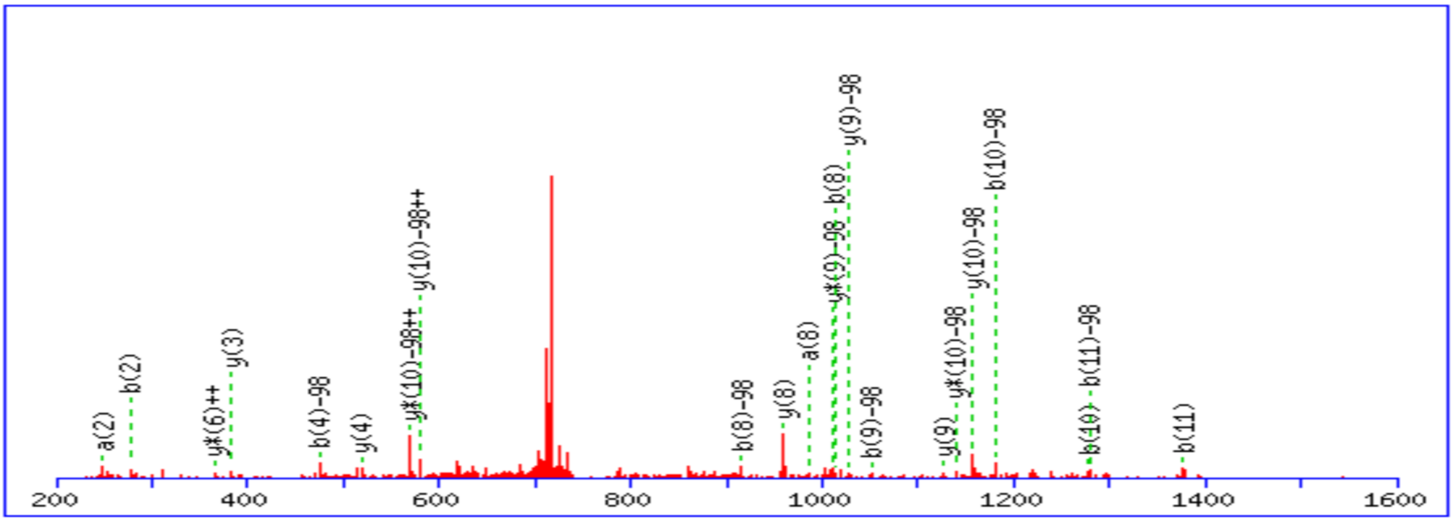

#	a	a++	a*	a*++	b	b++	b*	b*++	Seq.	y	y++	y*	y*++	#
1	136.0757	68.5415			164.0706	82.5389			Y					12
2	**249.1598**	125.0835			**277.1547**	139.0810			I	1270.6860	635.8466	1253.6595	627.3334	11
3	378.2023	189.6048			406.1973	203.6023			E	**1157.6019**	**579.3046**	**1140.5754**	**570.7913**	10
4	447.2238	224.1155			**475.2187**	238.1130			S	**1028.5594**	514.7833	**1011.5328**	506.2700	9
5	544.2766	272.6419			572.2715	286.6394			P	**959.5379**	480.2726	942.5114	471.7593	8
6	659.3035	330.1554			687.2984	344.1529			D	862.4851	431.7462	845.4586	423.2329	7
7	758.3719	379.6896			786.3668	393.6871			V	747.4582	374.2327	730.4316	**365.7195**	6
8	887.4145	444.2109			**915.4094**	458.2084			E	648.3898	324.6985	631.3632	316.1853	5
9	1023.5237	512.2655	1006.4971	503.7522	**1051.5186**	526.2629	1034.4921	517.7497	K	**519.3472**	260.1772	502.3206	251.6640	4
10	1152.5663	576.7868	1135.5397	568.2735	**1180.5612**	590.7842	1163.5346	582.2710	E	**383.2380**	192.1226	366.2115	183.6094	3
11	1251.6347	626.3210	1234.6081	617.8077	**1279.6296**	640.3184	1262.6031	631.8052	V	254.1954	127.6014	237.1689	119.0881	2
12									K	155.1270	78.0671	138.1005	69.5539	1

TAF1D; SRGSGFPFLESpENEK + Phospho (S)

Ion score: 38

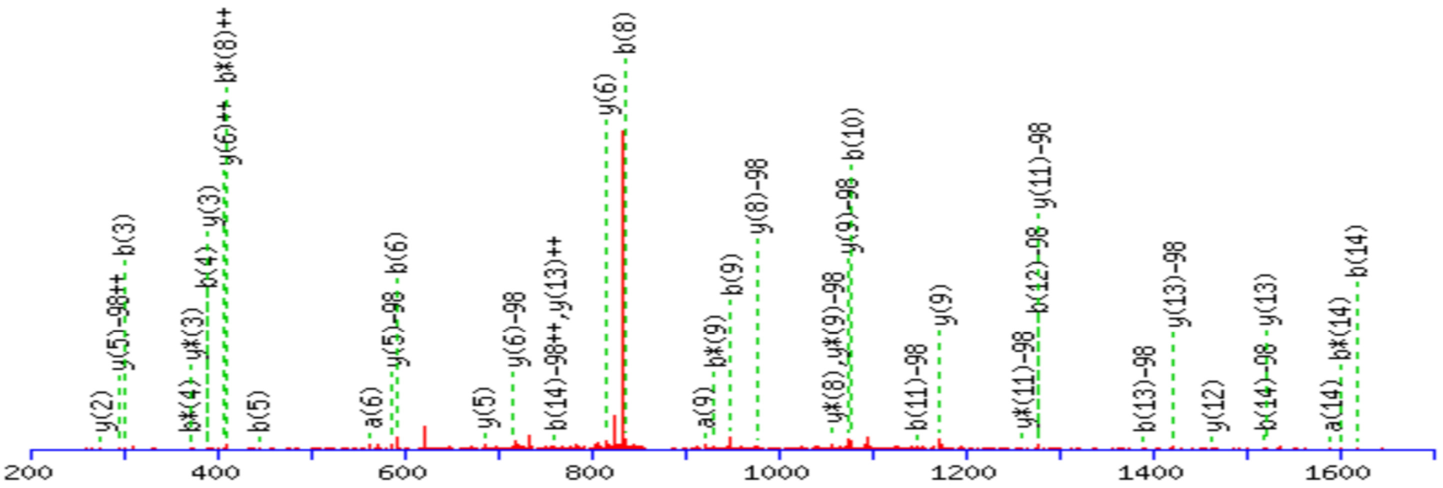

#	a	a++	a*	a*++	b	b++	b*	b*++	Seq.	y	y++	y*	y*++	#
1	60.0444	30.5258			88.0393	44.5233			S					15
2	216.1455	108.5764	199.1190	100.0631	244.1404	122.5738	227.1139	114.0606	R	1578.7547	789.8810	1561.7281	781.3677	14
3	273.1670	137.0871	256.1404	128.5738	**301.1619**	151.0846	284.1353	142.5713	G	**1422.6536**	711.8304	1405.6270	703.3172	13
4	360.1990	180.6031	343.1724	172.0899	**388.1939**	194.6006	**371.1674**	186.0873	S	1365.6321	683.3197	1348.6056	674.8064	12
5	417.2205	209.1139	400.1939	200.6006	**445.2154**	223.1113	428.1888	214.5980	G	**1278.6001**	639.8037	**1261.5735**	631.2904	11
6	**564.2889**	282.6481	547.2623	274.1348	**592.2838**	296.6455	575.2572	288.1323	F	1221.5786	611.2929	1204.5521	602.7797	10
7	661.3416	331.1745	644.3151	322.6612	689.3365	345.1719	672.3100	336.6586	P	**1074.5102**	537.7587	**1057.4837**	529.2455	9
8	808.4100	404.7087	791.3835	396.1954	**836.4050**	418.7061	819.3784	**410.1928**	F	**977.4574**	489.2324	960.4309	480.7191	8
9	**921.4941**	461.2507	904.4676	452.7374	**949.4890**	475.2482	**932.4625**	466.7349	L	830.3890	415.6982	813.3625	407.1849	7
10	1050.5367	525.7720	1033.5102	517.2587	**1078.5316**	539.7694	1061.5051	531.2562	E	**717.3050**	359.1561	700.2784	350.6428	6
11	1119.5582	560.2827	1102.5316	551.7694	**1147.5531**	574.2802	1130.5265	565.7669	S	**588.2624**	**294.6348**	571.2358	286.1216	5
12	1248.6008	624.8040	1231.5742	616.2907	**1276.5957**	638.8015	1259.5691	630.2882	E	519.2409	260.1241	502.2144	251.6108	4
13	1362.6437	681.8255	1345.6171	673.3122	**1390.6386**	695.8229	1373.6120	687.3097	N	**390.1983**	195.6028	**373.1718**	187.0895	3
14	1491.6863	746.3468	1474.6597	737.8335	**1519.6812**	**760.3442**	1502.6546	751.8310	E	**276.1554**	138.5813	259.1288	130.0681	2
15									K	147.1128	74.0600	130.0863	65.5468	1

TAF1D; TQCIPYSpPKGEK + Phospho (S)
Ion score: 36

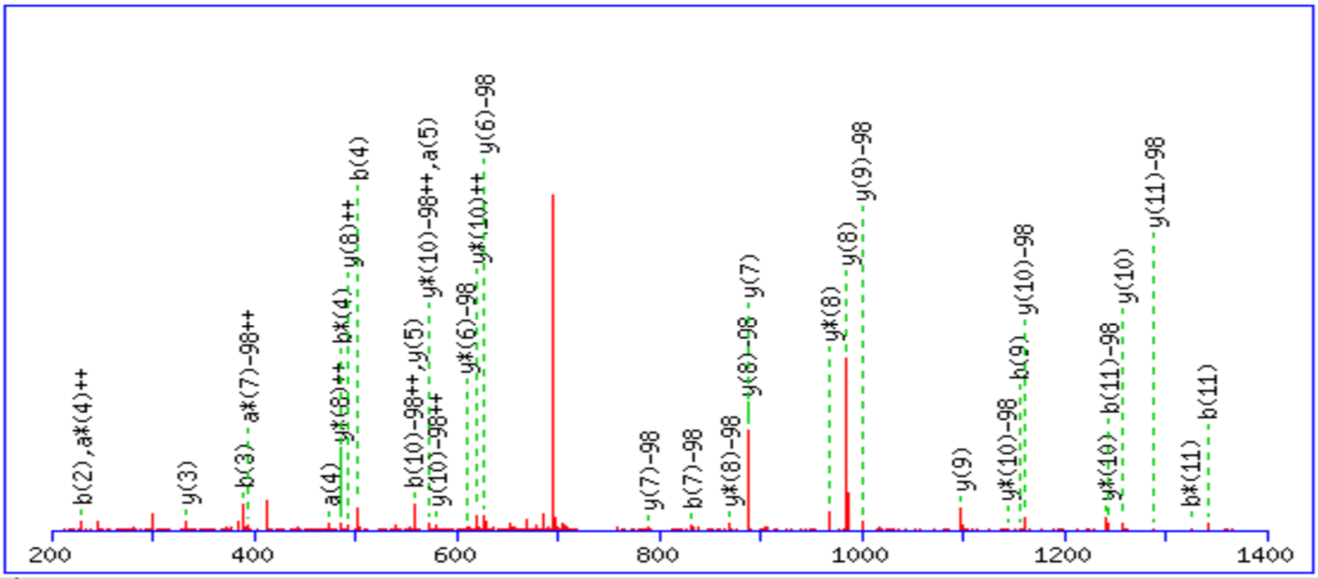

#	a	a^{++}	a*	a^{*++}	b	b^{++}	b*	b^{*++}	Seq.	y	y^{++}	y*	y^{*++}	#
1	74.0600	37.5337			102.0550	51.5311			T					12
2	202.1186	101.5629	185.0921	93.0497	**230.1135**	115.5604	213.0870	107.0471	Q	**1288.6354**	644.8214	1271.6089	636.3081	11
3	362.1493	181.5783	345.1227	173.0650	**390.1442**	195.5757	373.1176	187.0625	C	**1160.5769**	**580.7921**	**1143.5503**	**572.2788**	10
4	**475.2333**	238.1203	458.2068	**229.6070**	**503.2282**	252.1178	**486.2017**	243.6045	I	**1000.5462**	500.7767	983.5197	492.2635	9
5	**572.2861**	286.6467	555.2595	278.1334	600.2810	300.6441	583.2545	292.1309	P	**887.4621**	444.2347	**870.4356**	435.7214	8
6	735.3494	368.1784	718.3229	359.6651	763.3443	382.1758	746.3178	373.6625	Y	**790.4094**	395.7083	773.3828	387.1951	7
7	804.3709	402.6891	787.3443	**394.1758**	**832.3658**	416.6865	815.3392	408.1733	S	**627.3460**	314.1767	**610.3195**	305.6634	6
8	901.4236	451.2155	884.3971	442.7022	929.4186	465.2129	912.3920	456.6996	P	**558.3246**	279.6659	541.2980	271.1527	5
9	1029.5186	515.2629	1012.4921	506.7497	1057.5135	529.2604	1040.4870	520.7471	K	461.2718	231.1396	444.2453	222.6263	4
10	1086.5401	543.7737	1069.5135	535.2604	1114.5350	**557.7711**	1097.5084	549.2579	G	**333.1769**	167.0921	316.1503	158.5788	3
11	1215.5827	608.2950	1198.5561	599.7817	**1243.5776**	622.2924	1226.5510	613.7792	E	276.1554	138.5813	259.1288	130.0681	2
12									K	147.1128	74.0600	130.0863	65.5468	1
